# ﻿*Asterquanzhouensis* (Asteraceae), a new riparian species from eastern China

**DOI:** 10.3897/phytokeys.195.82411

**Published:** 2022-05-09

**Authors:** Jia-Wei Xiao, Guo-Jiao Yan, Wei-Ping Li, Ming Tang

**Affiliations:** 1 College of Urban and Rural Construction, Shaoyang University, Shaoyang, 422004, Hunan, China Shaoyang University Shaoyang China; 2 People’s Government of Jindou Town, Yongchun County, Quanzhou, 362613, Fujian, China People’s Government of Jindou Town Quanzhou China; 3 College of Life Sciences, Hunan Normal University, Changsha, 410081, Hunan, China Hunan Normal University Changsha China; 4 College of Forestry, Jiangxi Agricultural University, Nanchang 330045, Jiangxi, China Jiangxi Agricultural University Nanchang China

**Keywords:** Asteraceae, *Asterquanzhouensis*, new species, taxonomy

## Abstract

*Asterquanzhouensis***sp. nov.** (Asteraceae) from Fujian, eastern China, is described and illustrated. It grows on rocks in the riparian zone. Morphological, cytological and molecular investigations of *A.quanzhouensis* were carried out. The morphological data and phylogenetic analysis based on combined ITS, ETS and *trnL-F* dataset suggest that *A.quanzhouensis* is a separate species closely related to *A.tonglingensis*. The new species differs from the latter by the shorter stem length, leaf morphology, colour of phyllaries, number of ray florets, and achene shape. The cytological observation shows that the new species is diploid with a karyotype of 2n = 18.

## ﻿Introduction

The genus *Aster* L. in its recent circumscription is restricted to Eurasia and comprises ~ 150 species, of which 123 occur in China (Chen et al. 2011), a main diversity centre of *Aster* (Li et al. 2012). Recently, ten new *Aster* species have been described, and almost all these species have a narrow distribution pattern known from only one or two populations in different regions of China (Zhang et al. 2015, 2019; Li et al. 2017, 2020; Xiao et al. 2019a, b, 2020, 2021; Xiong et al. 2019).

Recently, Guo-Jiao Yan, a young amateur naturalist and one of the authors of this paper, collected some unique samples from the riversides of the Min and Jin rivers, Quanzhou city, Fujian, eastern China. The morphological, cytological and phylogenetic data show that the specimens represent an undescribed species, which is reported herein.

## ﻿Materials and methods

### ﻿Material collection

**Figure 1.**
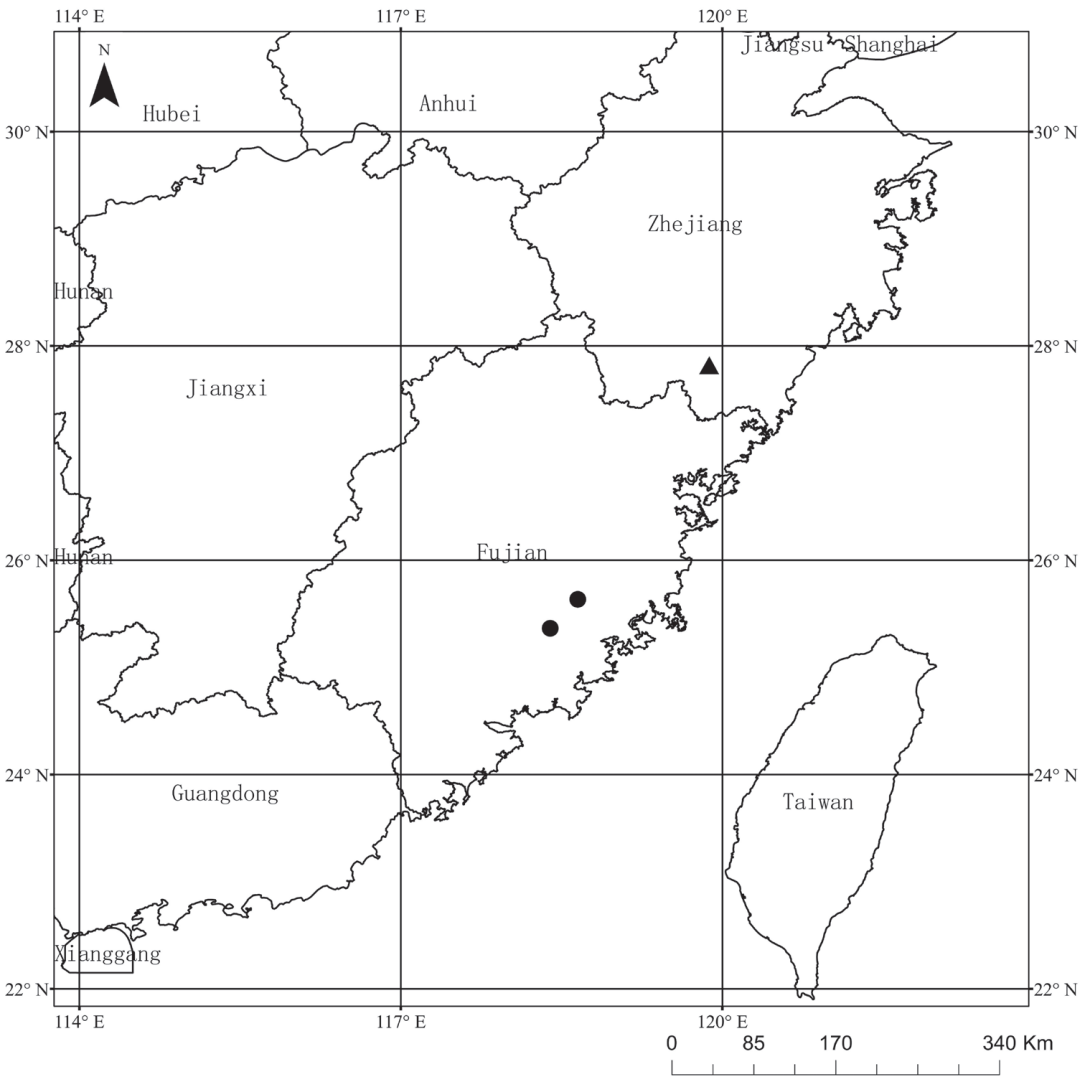
Distribution of *Asterquanzhouensis* (black circle) and *A.tonglingensis* (black triangle).

Specimens of the new taxon were collected in Dehua and Yongchun counties (Fig. 1), Fujian, China. We collected leaf material and dried it with silica gel for molecular experiments. The voucher specimens were deposited at the Herbarium of Hunan Normal University (HNNU) and Jiangxi Agricultural University (JXAU).

### ﻿Morphological observations

The description of the new species is based on living material, dry specimens and FAA-fixed materials. Twenty-one individuals were examined. The morphological comparison with *Astertonglingensis* G.J.Zhang & T.G.Gao is based on the study of herbarium specimens, from PE, HNNU and JAXU. We compared the shape and size of the leaves, length of stems, phyllaries, number of florets, and achenes.

### ﻿Cytology

Excised root tips from the cultivated plants of the new species were pretreated with 0.1% colchicine at 10 °C for 4 h, then fixed in Carnoy’s solution (95% ethanol and glacial acetic acid in 3:1 ratio) at 20 °C for 12 h. The root tips were then macerated in 1 M hydrochloric acid at 60 °C for 10 min, stained in Carbol fuchsin solution, washed in distilled water for 30 min and finally depigmented and squashed in 45% acetic acid (Li et al. 2011). Karyotype formulae were calculated based on measurements of mitotic metaphase chromosomes taken from photographs. The symbols used to describe the karyotypes followed Levan et al. (1964).

### ﻿Taxon sampling, DNA extraction, PCR reaction and sequencing

Nuclear ribosomal DNA ITS and ETS sequences and plastid DNA *trnL-F* sequences of 66 species and varieties, representing major clades of the genus *Aster* and its relatives (Li et al. 2012, 2017, 2020; Zhang et al. 2015, 2019; Xiao et al. 2019a, b, 2020, 2021), were downloaded from GenBank (Appendix 1). Besides, eleven newly sequenced accessions are included from Dehua and Yongchun counties two individuals of *Asterquanzhouensis* (Appendix 1). The names of the taxa mentioned above follow Chen et al. (2011). *Grangeamaderaspatana* (L.) Poir. and *Dichrocephalaintegrifolia* (L.f.) Kuntze were selected as outgroups following Li et al. (2012). Voucher specimens of newly sequenced material were deposited in HNNU. Total DNA extraction, PCR and sequencing were carried out according to Li et al. (2012).

### ﻿Phylogenetic analysis

Boundaries of the ITS, ETS and *trnL-F* regions were determined through comparison with previously published sequences (Li et al. 2012). DNA sequences were aligned initially using Clustal X1.83 (Jeanmougin et al. 1998), performed by MUSCLEv3.8.31 (Edgar 2004), and adjusted manually in PhyDE ver0.9971 (Müller et al. 2010). The optimal model of DNA substitutions was selected using the Akaike information criterion (Akaike 1973) as applied in jModelTest 2.1.4 (Darriba et al. 2012) prior to the maximum likelihood (ML) analyses and Bayesian inference (BI). The best fit models for ITS, ETS and *trnL-F* were GTR + G, GTR + I + G and TVM+I, respectively. Phylogenetic trees were constructed using maximum likelihood (ML) and Bayesian inference (BI). Maximum likelihood (ML) and Bayesian inference (BI) analyses were conducted using RAxML 7.2.6 and MrBayes 3.1.2 (Huelsenbeck and Ronquist 2001; Stamatakis 2006), respectively. For BI, four chains, each starting with a random tree, were run for 1,000,000 generations with trees sampled every 1000 generations. The average standard deviation of split frequencies (< 0.01) was used to assess the convergence of the two runs. After the first ca. 25% discarded as burn-in, the remaining trees were imported into PAUP* v.4.0b10 and a 50% majority-rule consensus tree was produced to obtain posterior probabilities (PP) of the clades. Before the datasets were combined, the incongruence length difference test (Farris et al. 1994) was performed on PAUP* v.4.0b10 (Swofford 2001).

## ﻿Results

### ﻿ Aster quanzhouensis

Taxon classificationPlantaeAsteralesAsteraceae

M.Tang, G.J.Yan & W.P.Li sp. nov.

E9396B43-12C0-59A1-90E9-86F0F7830DCF

urn:lsid:ipni.org:names:77297480-1

Figs 1 , 2 , 3

#### Type.

China, Fujian province, Quanzhou city, Dehua county, Nancheng town, alt. ca. 500 m, 25°34.20'N, 118°29.65'E, 5 Oct 2021, Guo-Jiao Yan, YGJ2110003 (Holotype: HNNU!, isotypes: HNNU!, JXAU!) (Fig. 3).

#### Additional collection seen.

China. Fujian province, Quanzhou city, Yongchun county, alt. ca. 500 m, 25°24'N, 118°21'E, 30 Nov 2021, Guo-Jiao Yan, YGJ21113001 (HNNU!).

#### Diagnosis.

**Figure 2.**
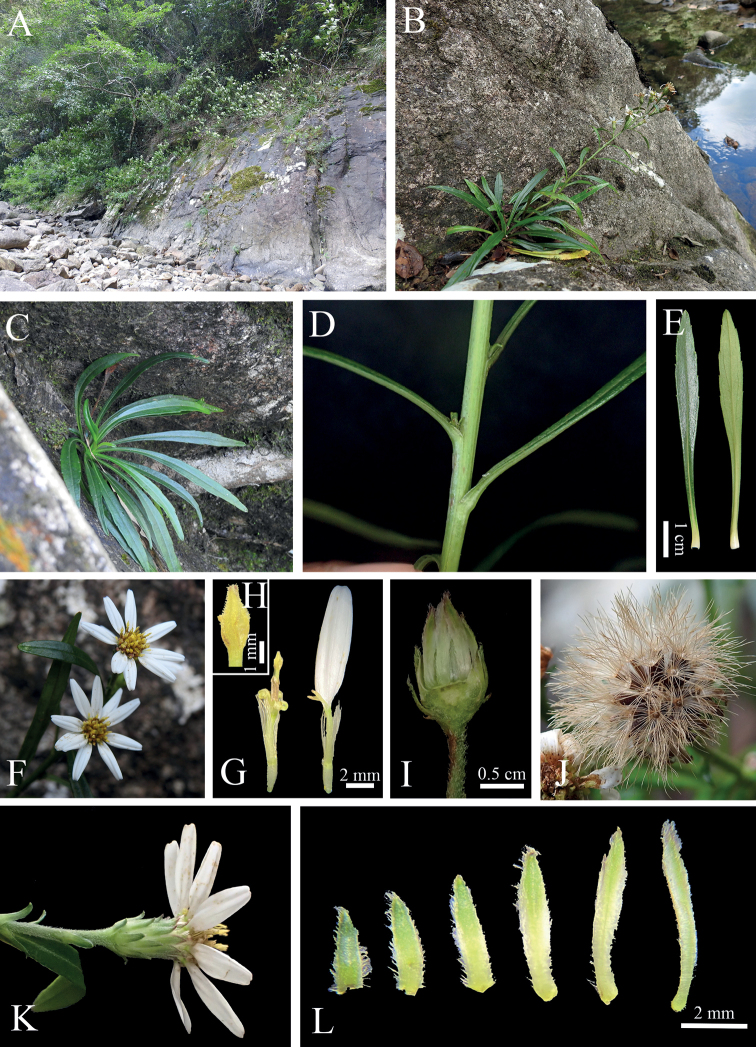
Images of living plants of *Asterquanzhouensis***A** habitat **B** habit **C** seedling **D** steam **E** rosulate leaves **F** top view of the capitulum **G** a disk floret (left) and a ray floret (right) **H** style branches of a disk floret **I** receptacle **J** fruits **K** dorsal view of a capitulum **L** phyllaries (from outer to inner, left to right).

**Figure 3.**
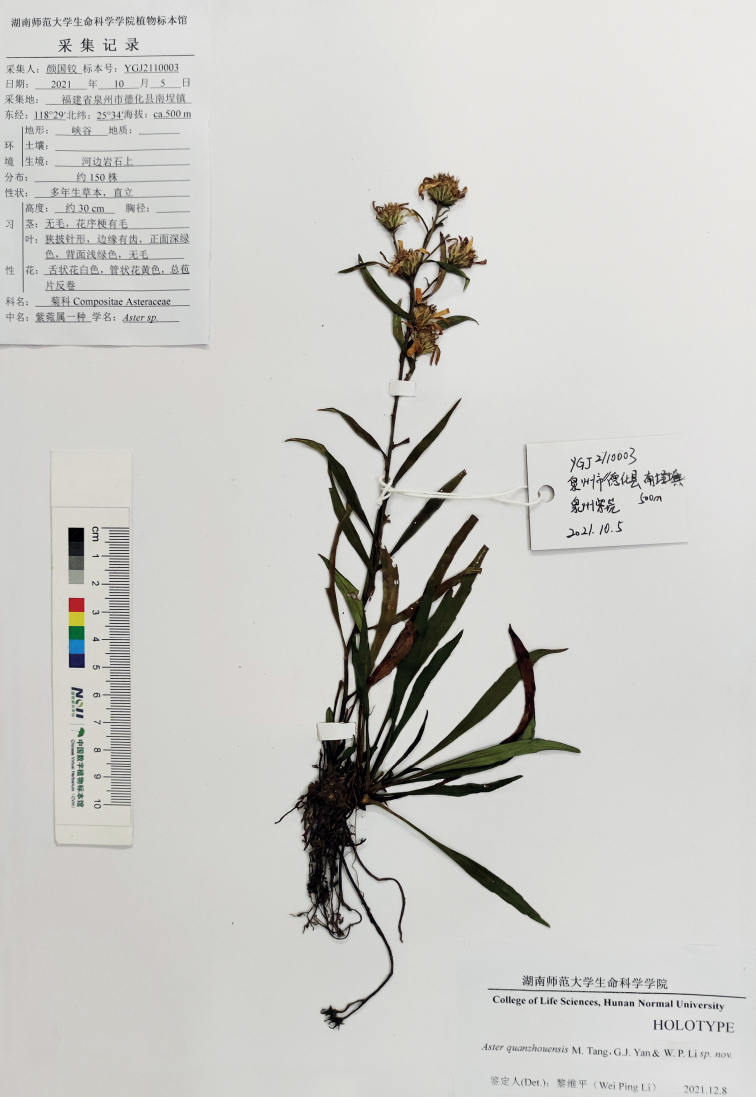
Holotype of *Asterquanzhouensis* M.Tang, G.J.Yan & W.P.Li.

*Asterquanzhouensis* differs from *A.tonglingensis* by its stems only 21–30 (60) cm (vs. 70–100 cm) long, narrowly lanceolate (vs. lanceolate) rosulate leaves, purplish-red (vs. green) apices of the phyllaries, 9–20 (40) (vs. more than 30) capitula, 7–11 (vs. ca. 15) ray florets, 11–14 × ca. 2 mm (vs. 7–10 × ca. 2 mm) lamina, two-or three-ribbed (vs. 4-ribbed) achenes and flowering period (Sep to early Dec vs. Jul) (Figs 2, 3, Table 1).

**Table 1.** Comparison of *Asterquanzhouensis* and *A.tonglingensis*. The data of the latter species were taken from Zhang et al. (2019).

Characters	*Asterquanzhouensis*	*A.tonglingensis*
**Stem**	21–30(60) cm, solitary	70–100 cm, solitary or two to three
**Basal leaves**	narrowly lanceolate, 4–13 × 0.4–1.7 cm	lanceolate, 4–18 × 0.8–2.5 cm
**Capitula**	9–20 (40)	More than 30
**Phyllaries**	5–7-seriate, apex purplish-red	5–7-seriate, apex green
**Ray florets**	7–11	ca. 15
**Achenes**	2–3-ribbed	4-ribbed
**Pappus**	8–11 mm	ca. 7 mm
**Flowering period**	Sep to early Dec	Jul

#### Description.

Perennial herb, 21–30 (60) cm high. Rhizomes thin, with adventitious roots, stolons absent. Stem solitary, erect, unbranched except for inflorescence, glabrous or puberulent in upper part. Leaves slightly leathery, narrowly lanceolate, apex acute, base gradually narrowing, subclasping, abaxially light green, adaxially dark green and glossy, 3-veined, midvein abaxially prominent; rosulate leaves 4–13 × 0.4–1.7 cm, margin serrate, petiole 3–11 cm long, both surfaces glabrous; lower cauline leaves persistent at anthesis or rarely withered, 3–10 × 0.3–0.8 cm, sessile or with obscure petioles, margin entire or serrate, abaxially glabrous, adaxially sparsely strigose; middle cauline leaves sessile or with obscure petiole, 4–7 × 0.3–0.4 cm, margin entire or serrate, abaxially glabrous, adaxially sparsely strigose; upper leaves sessile, margin entire. Capitula 9–20 (40) in a terminal corymbose cyme, peduncle puberulent. Involucre campanulate, 5–8 mm in diameter; phyllaries in 5–7 rows, imbricate, lanceolate, the outer rows shorter than the inner ones, reflexed, densely pilose, with ciliate margin; outer phyllaries 3.2–6.2 × 1.1–2 mm; middle phyllaries 4.6–13 × 1.5–2.2 mm, with narrowly scarious margin, tip purplish-red; inner phyllaries 10.1–13.0 ×1.5–1.7 mm, with broadly scarious margin, tip purplish-red. Receptacles flat, alveolate. Ray florets 7–11, female, tube ca. 4 mm, glabrous, ligules whitish, lanceolate, 11–14 × ca. 2 mm, with four nerves, apex with two or three teeth. Disc florets (11) 18–24, hermaphrodite, yellow, tube puberulent, ca. 3 mm, thin but expanded at base, 5-lobed, lobes spreading to reflexed, narrowly triangular, unequal, 1.1–1.5 mm, glandular; anthers ca. 1.8 mm (excluding collar), apical appendage 0.35–0.45 mm long, narrowly lanceolate, anther collar ca. 0.4 mm long; style arm appendage lanceolate, ca. 2.5 mm, stigmatic lines 1.2–1.4 mm, equal to the sterile style tip appendages. Achenes 4.5–5.5 × 0.9–1.4 mm, narrowly oblong, strigose, eglandular, two- or three-ribbed. Pappus uniseriate, dirty white, 8–11 mm, nearly as long as disc corolla at anthesis.

#### Phenology.

Flowering from September to early December and fruiting from October to December.

#### Etymology.

The species is named after its type locality, Quanzhou city, Fujian province, China.

#### Vernacular name.

quán zhōu zĭ wăn (Chinese pronunciation); 泉州紫菀 (Chinese name).

#### Distribution and habitat.

*Asterquanzhouensis* is known from Dehua and Yongchun counties, Quanzhou city, Fujian province, China. The new species grows on rocks in riparian habitats at an altitude of ca. 500 m a.s.l.

#### Conservation status.

*Asterquanzhouensis* seems to be a narrowly distributed species, currently known only in rocky areas along two streams (Jin river and Min river) in Quanzhou city, and each population with ca. 150 (total < 1000) individuals were found. The habitat of *A.quanzhouensis* is easily disturbed or damaged. Further fieldwork is needed to evaluate the exact distribution of the species, and it is possible that other populations could be found in similar habitats of the Jin and Min rivers. Therefore, we only temporarily assign the species to the category DD (Data Deficient) according to the International Union for Conservation of Nature (IUCN 2022).

##### ﻿Cytology

**Figure 4.**
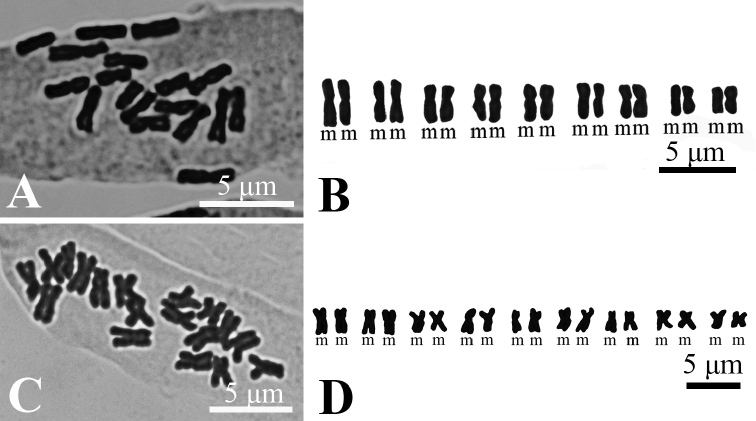
Micrographs of somatic metaphase chromosomes (**A, C**) and the karyotype (**B, D**) of *Asterquanzhouensis* from two different populations. (**A, B** Dehua county, Quanzhou, Fujian, China; **C, D** Yongchun county, Quanzhou, Fujian, China).

The somatic chromosomes of the new species at metaphase are illustrated in Fig. 4. The two populations have a same karyotype formula, 2n = 18, and Stebbins’ 1A-type (Stebbins 1971), but differs in ratio of long to short arm of chromosomes (the former is 1.02–1.55, while the latter 1.06–1.45), the chromosomes length (the former is 1.49–2.72, while the latter 1.71–2.77), and the AI value (the former is 0.54, while the latter 0.57).

##### ﻿Molecular phylogeny

**Figure 5.**
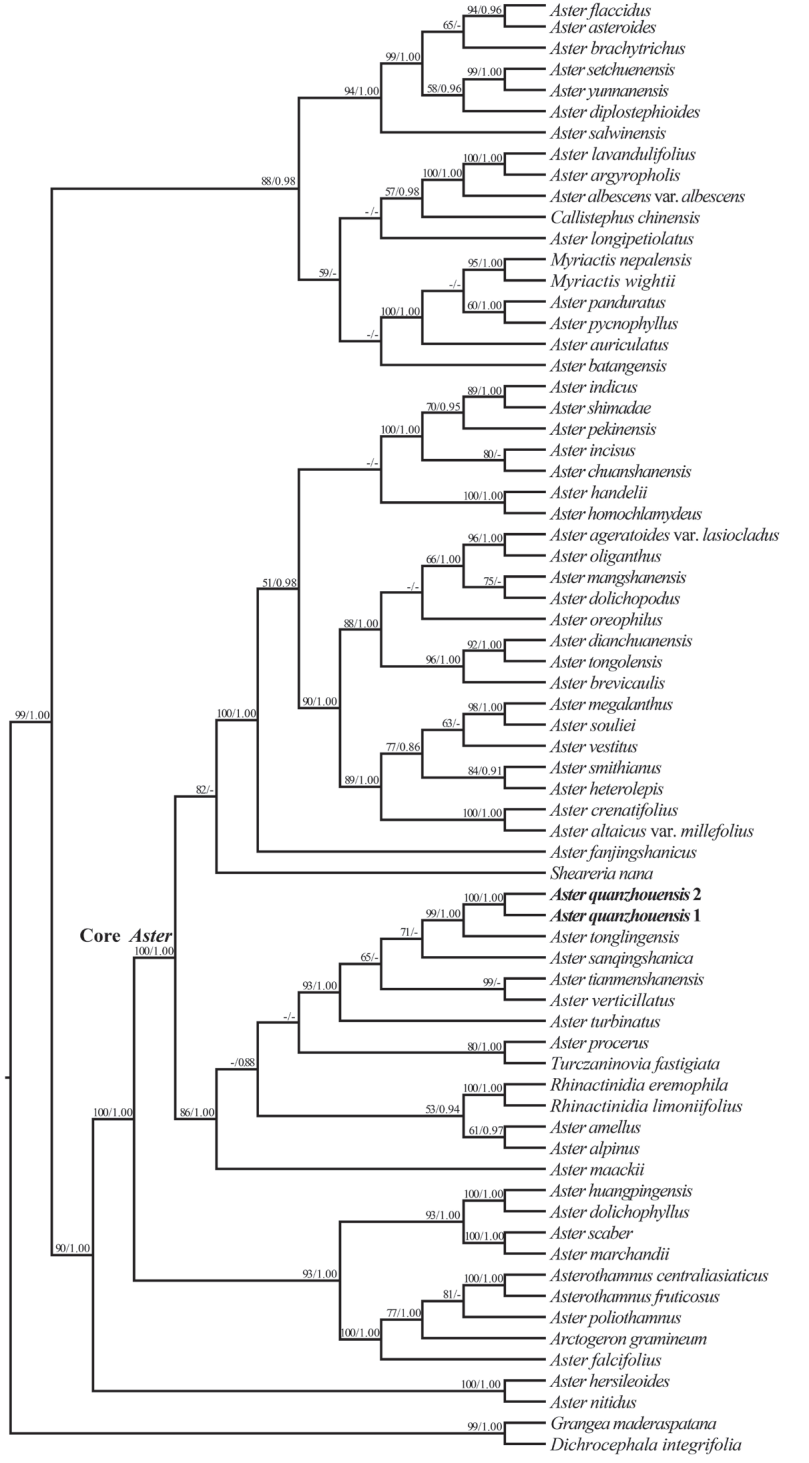
The phylogram of the maximum likelihood (ML) tree from the combined data (ITS, ETS and *trnL-F*), showing the phylogenetic position of *Asterquanzhouensis*. Bootstrap support values (1,000 replicates) for maximum parsimony (MP ≥ 50%, left) and Bayesian posterior probabilities (PP ≥ 0.90, right) are provided above the branches. The samples of *Asterquanzhouensis* are shown in bold.

The aligned lengths of ITS, ETS and *trnL-F* are 647 bp, 568 bp and 957 bp, respectively, yielding a concatenated alignment of 2172 bp. Character state changes were equally weighted and gaps were treated as missing data. ML and BI analyses produced similar topology and only the ML tree was presented in Fig. 5, with ML bootstrap (LP), and PP values for each clade. The phylogenetic results showed that the two samples of the new taxon were grouped together with strong support (PP = 1.00, LP = 100%) and are closely related to *Astertonglingensis* with strong support (PP = 1.00, LP = 99%). According to these results, *A.quanzhouensis* is nested within the core *Aster* clade (PP = 1.00, LP = 100%) that is the redefined genus *Aster* in Eurasia (Li et al. 2012; Nesom 2020a, b).

## ﻿Discussion

Morphological observations showed that *Asterquanzhouensis* has a perennial life form, lanceolate stigmatic appendage of disc florets, compressed fruits with two- or three-ribbed and uniseriate pappus (Figs 2, 3). All *Aster* species share these characters. In the phylogenetic tree (Fig. 5), *A.quanzhouensis* is deeply nested within the core *Aster* (Li et al. 2012). Morphological and phylogenetic analyses support that *A.quanzhouensis* is sister to *A.tonglingensis*. As mentioned above, the two species can be easily distinguished from each other (Figs 2, 3, Table 1).

Narrowly lanceolate leaves are rare in Eurasian *Aster* and can be found only in a few species, such as *A.huangpingensis* W.P.Li & Z.Li, *A.dolichophyllus* Y.Ling and *A.tonglingensis*. Our phylogenetic analyses (Fig. 5) revealed that the species with narrowly lanceolate leaves are nested in unrelated lineages of the genus *Aster* and are probably the result of convergent evolution. It is noteworthy to mention that they are all distributed in the same habitats confined to riparian rocks (Chen et al. 2011; Zhang et al. 2019; Li et al. 2020). The same leaf character may be related to their habitat. When the water level rises in some periods during the course of the year, these species were submerged and their narrowly lanceolate leaves may represent adaptation to water flowing in the rivers or streams.

Karyotype variation usually accompanies evolutionary divergence, a general phenomenon observed in plants and animals (Rieseberg 2001). Two populations of the *Asterquanzhouensis* were found with the same karyotype formula and Stebbins’ type, with only slight differences in the karyotypic indexes, which might mean that *A.quanzhouensis* is a young species.

*Asterquanzhouensis* is known only from two populations (Dehua and Yongchun counties) restricted to Quanzhou, Fujian, China, while *A.tonglingensis* is restricted to Mt. Tongling Natural Reserve, Wencheng county, Zhejiang (Zhang et al. 2019). These two species occupy the same ecological conditions, but are geographically separated by a distance of 400 km.

## Supplementary Material

XML Treatment for Aster quanzhouensis

## ﻿Appendix 1

**Table A1.** Taxa sampled, vouchers and GenBank accessions. The newly sequenced samples are highlighted in bold.

Accessions	Voucher information or references	Accession number		
		ITS	ETS	*trnL-F*
***Asterquanzhouensis* M.Tang, G.J.Yan & W.P.Li 1**	*Guo-Jiao Yan*, *YGJ2110001*, Dehua county, Fujian, China	ON055150	ON055152	ON055154
***Asterquanzhouensis* M.Tang, G.J.Yan & W.P.Li 2**	*Guo-Jiao Yan*, *YGJ2112001*, Yongchun county, Fujian, China	ON055151	ON055153	ON055155
***A.sanqingshanica*** J.W.Xiao & W.P.Li	Xiao et al. (2021)	MW419955	MW419952	ON055156
***A.marchandii*** H.Lév.	Xiao et al. (2021)	MW419957	MW419954	ON055157
*A.dianchuanensis* J.W.Xiao & W.P.Li	Xiao et al. (2019b)	MK693180	MK693190	MK693202
*A.brevicaulis* W.P.Li	Xiao et al. (2019a)	MH638204	MH638209	MH638218
*A.tongolensis* Franch.	Xiao et al. (2019b)	MK693183	MK693193	JN543834
A.ageratoidesvar.lasiocladus (Hayata) Hand.-Mazz.	Li et al. (2012)	JN543781	JN543782	JN543783
*A.oliganthus* W.P.Li & Z.Li	Li et al. (2017)	KY428860	KY428852	MH638219
*A.mangshanensis* Y.Ling	Li et al. (2012)	JN543760	JN543761	JN543762
*A.oreophilus* Franch.	Li et al. (2012)	JN543826	JN543827	JN543828
*A.dolichopodus* Y.Ling	Li et al. (2012)	JN543775	JN543776	JN543777
*A.vestitus* Franch.	Li et al. (2012)	JN543769	JN543770	JN543771
*A.souliei* Franch.	Li et al. (2012)	JN543835	JN543836	JN543837
*A.megalanthus* Y.Ling	Xiao et al. (2019b)	MK693187	MK693197	MK693207
*A.smithianus* Hand.-Mazz.	Li et al. (2012)	JN543778	JN543779	JN543780
*A.heterolepis* Hand.-Mazz.	Li et al. (2012)	JN543823	JN543824	JN543825
A.altaicusvar.millefolius (Vaniot) Hand.-Mazz.	Li et al. (2012)	JN543709	JN543710	JN543711
*A.crenatifolius* Hand.-Mazz.	Li et al. (2012)	JN543712	JN543713	JN543714
*A.fanjingshanicus* Y.L.Chen & D.J.Liu	Li et al. (2012)	JN543829	JN543830	JN543831
*A.pekinensis* (Hance) F.H.Chen	Li et al. (2012)	JN543718	JN543719	JN543720
***A.shimadae*** (Kitamura) Nemoto	Xiao et al. (2020)	MT731682	MT731599	ON055158
*A.indicus* L.	Li et al. (2012)	JN543715	JN543716	JN543717
*A.incisus* Fisch.	Li et al. (2012)	JN543721	JN543722	JN543723
***A.chuanshanensis*** W.P.Li	Xiao et al. (2020)	MT731676	MT731593	ON055159
*A.homochlamydeus* Hand.-Mazz.	Li et al. (2012)	JN543784	JN543785	JN543786
*A.handelii* Onno	Li et al. (2012)	JN543820	JN543821	JN543822
*A.maackii* Regel	Li et al. (2012)	JN543745	JN543746	JN543747
*A.turbinatus* S.Moore	Li et al. (2012)	JN543814	JN543815	JN543816
*A.verticillatus* (Reinw.) Brouillet	Li et al. (2012)	JN543706	JN543707	JN543708
*A.tianmenshanensis* G.J.Zhang & T.G.Gao	Zhang et al. (2015)	KP313677	KP313690	KP313703
*A.tonglingensis* G.J.Zhang & T.G.Gao	Zhang et al. (2019)	MH807119	MH807124	MH807126
*A.procerus* Hemsl.	Zhang et al. (2015)	KP313683	KP313696	KP313709
*A.amellus* Grierson	Li et al. (2012)	JN543742	JN543743	JN543744
*A.alpinus* L.	Li et al. (2012)	JN543817	JN543818	JN543819
*A.falcifolius* Hand.-Mazz.	Li et al. (2012)	JN543802	JN543803	JN543804
*A.poliothamnus* Diels	Li et al. (2012)	JN543763	JN543764	JN543765
*A.scaber* Thunb.	Li et al. (2012)	JN315934	JN315958	JN315910
***A.huangpingensis*** W.P.Li & Z.Li	Li et al. (2020)	MH747070	MH747071	ON055160
*A.dolichophyllus* Y.Ling	Zhang et al. (2019); Li et al. (2020)	MH747068	MH747069	MH807108
*A.hersileoides* C.K.Schneid.	Li et al. (2012)	JN543787	JN543788	JN543789
*A.nitidus* C.C.Chang	Li et al. (2012)	JN543790	JN543791	JN543792
*A.salwinensis* Onno	Zhang et al. (2015)	KP313689	KP313702	KP313715
*A.diplostephioides* (DC.) Benth. ex C.B.Clarke	Li et al. (2012)	JN543847	JN543848	JN543849
*A.setchuenensis* Franch.	Li et al. (2012)	JN543850	JN543851	JN543852
*A.yunnanensis* Franch.	Li et al. (2012)	JN543853	JN543854	JN543855
*A.brachytrichus* Franch.	Li et al. (2012)	JN543838	JN543839	JN543840
*asteroides* (DC.) Kuntze	Li et al. (2012)	JN543841	JN543842	JN543843
*A.flaccidus* Bunge	Li et al. (2012)	JN543844	JN543845	JN543846
*A.batangensis* Bureau & Franch.	Li et al. (2012)	JN543859	JN543860	JN543861
*A.panduratus* Nees ex Walp.	Li et al. (2012)	JN543757	JN543758	JN543759
*A.auriculatus* Franch.	Li et al. (2012)	JN543754	JN543755	JN543756
*A.pycnophyllus* Franchet ex W.W.Sm.	Li et al. (2012)	JN543799	JN543800	JN543801
*A.longipetiolatus* C.C.Chang	Li et al. (2012)	JN315936	JN315960	JN315912
*A.lavandulifolius* Hand.-Mazz.	Li et al. (2012)	JN543796	JN543797	JN543798
*A.argyropholis* Hand.-Mazz.	Li et al. (2012)	JN543793	JN543794	JN543795
A.albescens (DC.) Wall. ex Hand.-Mazz. var. albescens	Li et al. (2012)	JN543862	JN543863	JN543864
*Shearerianana* S.Moore	Li et al. (2012)	JN543703	JN543704	JN543705
*Arctogerongramineum* (L.) DC.	Li et al. (2012)	JN315928	JN315952	JN315904
*Asterothamnusfruticosus* (C.Winkl.) Novopokr.	Li et al. (2012)	JN315929	JN315953	JN315905
*A.centraliasiaticus* Novopokr.	Li et al. (2012)	JN315930	JN315954	JN315906
*Callistephuschinensis* (L.) Nees	Li et al. (2012)	JN315931	JN315955	JN315907
*Myriactiswightii* DC.	Li et al. (2012)	JN315922	JN315946	JN315898
*M.nepalensis* Less.	Li et al. (2012)	JN315921	JN315945	JN315897
*Rhinactinidialimoniifolia* (Less.) Novopokr. ex Botsch.	Li et al. (2012)	JN543724	JN543725	JN543726
*Rh.eremophila* (Bunge) Novopokr. ex Botsch.	Li et al. (2012)	JN543727	JN543728	JN543729
*Turczaninoviafastigiata* (Fisch.) DC	Li et al. (2012)	JN543739	JN543740	JN543741
*Grangeamaderaspatana* (L.f.) Kuntze	Li et al. (2012)	JN315920	JN315944	JN315896
*Dichrocephalaintegrifolia* (L.) Poir.	Li et al. (2012)	JN315919	JN315943	JN315895

## References

[B1] Akaike H (1973) Information theory and an extension of the maximum likelihood principle. In: Petrov BN Caski F (Eds) Proceedings of the Second International Symposium on Information Theory.Akademiai, Kiado Budapest, 267–281.

[B2] Chen YL Brouillet L Semple JC (2011) *Aster*. In: Wu ZY Raven PH Hong DY (Eds) Flora of China Vol.20–21. Science Press, Beijing & Missouri Botanical Garden Press, St. Louis, 574–632.

[B3] Darriba D Taboada GL Doallo R Posada D (2012) jModelTest 2: More models, new heuristics and parallel computing. Nature Methods 9(8): e772. 10.1038/nmeth.2109 PMC4594756 22847109

[B4] Edgar RC (2004) MUSCLE: Multiple sequence alignment with high accuracy and high throughput. Nucleic Acids Research 32(5): 1792–1797. 10.1093/nar/gkh340 15034147 PMC390337

[B5] Farris SJ Kalersjo M Kluge AG Bult C (1994) Testing significance of incongruence. Cladistics 10(3): 315–319. 10.1111/j.1096-0031.1994.tb00181.x

[B6] Huelsenbeck JP Ronquist F (2001) MRBAYES: Bayesian inference of phylogenetic trees. Bioinformatics (Oxford, England) 17(8): 754–755. 10.1093/bioinformatics/17.8.754 11524383

[B7] IUCN (2022) Guidelines for Using the IUCN Red List Categories and Criteria. Version 15. Prepared by the Standards and Petitions Committee. http://www.iucnredlist.org/documents/RedListGuidelines.pdf

[B8] Jeanmougin F Thompson JD Gouy M Higgins DG Gibson TJ (1998) Multiple sequence alignment with Clustal X. Trends in Biochemical Sciences 23(10): 403–405. 10.1016/S0968-0004(98)01285-7 9810230

[B9] Levan A Fredga K Sandberg AA (1964) Nomenclature for centromeric position on chromosomes. Hereditas 52(2): 201–220. 10.1111/j.1601-5223.1964.tb01953.x

[B10] Li WP Tang M Yin GS Yin Y Yang FS Chen SM (2011) Extensive chromosome number variation in Asterageratoidesvar.pendulus (Asteraceae). Botanical Journal of the Linnean Society 165(4): 378–387. 10.1111/j.1095-8339.2011.01114.x

[B11] Li WP Yang FS Jivkova T Yin GS (2012) Phylogenetic relationships and generic delimitation of Eurasian *Aster* (Asteraceae: Astereae) inferred from ITS, ETS and *trnL-F* sequence data. Annals of Botany 109(7): 1341–1357. 10.1093/aob/mcs054 22517812 PMC3359916

[B12] Li Z Yin GS Tang M Li WP (2017) *Asteroliganthus* (Asteraceae, Astereae), a new species from western Sichuan, China, based on morphological and molecular data. Phytotaxa 326(1): 054–062. 10.11646/phytotaxa.326.1.4

[B13] Li Z Xiong YC Liao JJ Xiao JW Li WP (2020) *Asterhuangpingensis* (Asteraceae, Astereae), a new species from Guizhou, China. Phytotaxa 433(3): 235–244. 10.11646/phytotaxa.433.3.5

[B14] Müller K Müller J Quandt D (2010) PhyDE-Phylogenetic Data Editor, version 0.9971.

[B15] Nesom GL (2020a) Revised subtribal classification of Astereae (Asteraceae). Phytoneuron 53: 1–39.

[B16] Nesom GL (2020b) The genus *Aster* (Asteraceae) in the strictest sense. Phytoneuron 56: 1–22.

[B17] Rieseberg LH (2001) Chromosomal rearrangements and speciation. Trends in Ecology & Evolution 16(7): 351–358. 10.1016/S0169-5347(01)02187-5 11403867

[B18] Stamatakis A (2006) RAxML-VI-HPC: Maximum likelihood-based phylogenetic analyses with thousands of taxa and mixed models. Bioinformatics (Oxford, England) 22(21): 2688–2690. 10.1093/bioinformatics/btl446 16928733

[B19] Stebbins GL (1971) Chromosomal evolution in higher plants. Edward Arnold, London, 216 pp.

[B20] Swofford DL (2001) PAUP*: phylogenetic analysis using parsimony (*and other methods), version 4.0b10. Sinauer Associates, Sunderland, MA.

[B21] Xiao JW Liao JJ Li WP (2019a) *Asterbrevicaulis* (Asteraceae, Astereae), a new species from western Sichuan, China. Phytotaxa 399(1): 001–013. 10.11646/phytotaxa.399.1.1

[B22] Xiao JW Liao JJ Li WP (2019b) *Asterdianchuanensis* (Asteraceae, Astereae), a new species from Yunnan and Sichuan, China. Kew Bulletin 74(3): e50. 10.1007/s12225-019-9838-x

[B23] Xiao JW Zhao QY Xiong YC Li WP (2020) *Asterchuanshanensis* (Asteraceae), a New Species from Shanxi and Sichuan, China. Annales Botanici Fennici 57(4–6): 341–350. 10.5735/085.057.0417

[B24] Xiao JW Zhao QY Xie D Tang ZB Li WP (2021) *Astersanqingshanica* (Asteraceae, Astereae), a new species from Jiangxi, China. Nordic Journal of Botany 39(4): e02990. 10.1111/njb.02990

[B25] Xiong YC Li Z Xiao JW Li WP (2019) *Astersaxicola* sp. nov. (Asteraceae, Astereae), a new species from Guizhou, China: Evidence from morphological, molecular and cytological studies. Nordic Journal of Botany 37(10): e02364. 10.1111/njb.02364

[B26] Zhang GJ Hu HH Zhang CF Tian XJ Peng H Gao TG (2015) Inaccessible biodiversity on limestone cliffs: *Astertianmenshanensis* (Asteraceae), a new critically endangered species from China. PLoS ONE 10(8): 1–16. 10.1371/journal.pone.0134895 PMC4550265 26308863

[B27] Zhang GJ Hu HH Gao TG Gilbert MG Jin XF (2019) Convergent origin of the narrowly lanceolate leaf in the genus *Aster*-with special reference to an unexpected discovery of a new *Aster* species from East China. PeerJ 7: e6288. 10.7717/peerj.6288 PMC6348959 30701132

